# Systematic Review of Studies on Subliminal Exposure to Phobic Stimuli: Integrating Therapeutic Models for Specific Phobias

**DOI:** 10.3389/fnins.2021.654170

**Published:** 2021-06-02

**Authors:** Sergio Frumento, Danilo Menicucci, Paul Kenneth Hitchcott, Andrea Zaccaro, Angelo Gemignani

**Affiliations:** ^1^Department of Surgical, Medical, Molecular and Critical Area Pathology, University of Pisa, Pisa, Italy; ^2^National Research Council, Institute of Clinical Physiology, Pisa, Italy; ^3^Azienda Ospedaliero-Universitaria Pisana, Dipartimento di Specialità cliniche, Pisa, Italy

**Keywords:** phobia, subliminal, exposure therapy, anxiety disorders, skin conductance, desensitization, masked, unconscious

## Abstract

We systematically review 26 papers investigating subjective, behavioral, and psychophysiological correlates of subliminal exposure to phobic stimuli in phobic patients. Stimulations were found to elicit: (1) cardiac defense responses, (2) specific brain activations of both subcortical (e.g., amygdala) and cortical structures, (3) skin conductance reactions, only when stimuli lasted >20 ms and were administered with intertrial interval >20 s. While not inducing the distress caused by current (supraliminal) exposure therapies, exposure to subliminal phobic stimuli still results in successful extinction of both psychophysiological and behavioral correlates: however, it hardly improves subjective fear. We integrate those results with recent bifactorial models of emotional regulation, proposing a new form of exposure therapy whose effectiveness and acceptability should be maximized by a preliminary subliminal stimulation. Systematic Review Registration: identifier [CRD42021129234].

## Introduction

### Rationale

Specific phobias are a prototypical example of emotional dysregulation, being characterized by marked and disproportionate fear for specific objects or situations (American Psychiatric Association, 2013). They are traditionally considered a product of conditioning, both classical and operant. Learning by classical conditioning usually needs repeated pairing of stimuli, but a highly-emotional stimulus can trigger the learning process in a single event; learning by operant conditioning is based of rewards and punishments associated (or thought to be associated) with a behavior. According to Mowrer's 2-factor model (Mowrer, 1947, 1956), phobias are acquired through classical conditioning (e.g., snake-related trauma) and subsequently maintained through operant conditioning (e.g., avoiding woods to reduce the possibility to meet snakes, and getting relieved by such avoidance). Also, the Seligman's Theory of Preparedness (Seligman, 1971) described specific phobias in terms of conditioning, but incorporated the notion that some stimuli are more conditionable than others, thus providing a convenient explanation for the high incidence of specific phobias concerning spiders and snakes. Experimental demonstrations, that certain associations are more readily acquired depending on their evolutionary significance (Garcia and Koelling, 1966) supported this view. More recently, LeDoux (1994) proposed that fear responses are processed through different pathways in the brain: a subcortical pathway providing quick-and-dirty information, and a cortical pathway accounting for a more detailed analysis. He also suggested that both pathways could be malfunctioning in phobias, however current therapies only affect the cortical pathway.

Conditioning-based interventions based on *in vivo* exposure are the gold standard treatments for specific phobias (Choy et al., 2007): patients are exposed to the phobic stimulus with the aim of extinguishing the acquired phobic response. To facilitate this process, presentation of the phobic stimulus is often graded and/or combined with guided relaxation techniques. This approach has proven to be effective, but it also presents some disadvantages: patients have to remain in the presence of the phobic stimulus until the phobic response has fully recovered, but the more fearful patients are not able to face the stimulus for so long (Choy et al., 2007). This has the paradoxical consequence that only a minority of phobic patients seeks psychological help for specific phobia (Stinson et al., 2007): among these, a relevant rate of unresponsiveness to treatment (Loerinc et al., 2015) and dropouts (Eaton et al., 2018) has been reported. In fact, phobic patients have shown deficits in basic mechanisms (e.g., inhibitory learning) supposed to underlie exposure therapies, whose benefits can be maximized by adopting strategies that counterbalance patient's pathological functioning (Craske et al., 2014).

Subliminal exposure to the phobic stimulus represents the gold standard for studying the basic mechanisms of emotional regulation, due to its ability to distinguish behavioral and physiological correlates from subjective ones. There is no agreement on how to define and measure subliminal exposure (Wiens, 2006, 2007), nor in the proper term to use. In the scientific literature, many terms or phrases are used (e.g., unconscious, unreportable, covert, masked, sub-threshold, as well as automatic processing, very-brief-exposure, etc.) to indicate methods that are mostly comparable, mainly differing for the underlying theories and for the inferences proposed (e.g., both terms sub-threshold and subliminal imply the existence of a threshold). In this paper we will use the term subliminal stimuli, intended as those stimuli whose conscious perception is not subjectively reported by the subjects.

Over time, various techniques to make a stimulus subliminal have been developed. Most of masking methods (e.g., backward or forward masking, continuous flash suppression, very brief exposure) are based on a reduced duration of stimulus exposure and/or on a manipulation of attentional focus (Wiens, 2006). Backward masking was the most used paradigm, since its capability to keep the stimulus subliminal for a longer time (up to 33.4 ms, among the included papers) with respect to paradigms that show unmasked stimuli (Wiens, 2006). Differences in backward masking properties (e.g., stimulus duration) are detailed in **Table 3** and furtherly discussed whenever they can be reasonably thought to affect the results (e.g., for what concerns negative results in skin conductance responses to subliminal phobic stimuli, discussed in section Is Skin Conductance Activity a Reliable Marker of Phobic Subliminal Stimulation?). A minority of the included papers (Carretié et al., 2005; Granado et al., 2007; Schmack et al., 2016; Taschereau-Dumouchel et al., 2018a) used paradigms that do not share comparable features with the others: consequently, they are described in detail in section Experimental Paradigms. Among those, the Decoded Neural-Reinforcement technique based on neurofeedback (Taschereau-Dumouchel et al., 2018a) represents an exception within the exception, since it does not involve any direct exposure to the phobic stimulus (as detailed in section Experimental Paradigms) and should not be strictly considered as a subliminal stimulation: however, it fully falls into the selection criteria of the present review, since it claim to reach desensitization-like effects without exposing phobic patients to a consciously perceptible phobic stimulus.

The feasibility of therapeutic approach based on subliminal stimulations was suggested in a seminal paper by Öhman and Soares (1994) showing that this approach elicits—and subsequently, inhibits—a phobic reaction to subliminal stimuli. However, following studies failed to replicate their results, this leading to an unresolved debate (Mayer et al., 1999b; Öhman, 1999). Methodological heterogeneity exists among studies, and when experimental protocols are compared, some variables—such as stimulus duration and intertrial interval—appear to be possible critical determinants of the observed different results. Also, some studies have evaluated the effect of subliminal stimuli-based therapeutic approaches based on different outcomes: subjective (i.e., perceived fear), behavioral (capability to limit avoidance response) or psychophysiological (e.g., heart rate, skin conductance responses, brain activations) ones. All these factors may contribute to the contrasting conclusions reported in the different studies.

### Objectives and Research Questions

The aim of this paper is to systematically review the scientific literature concerning subliminal presentation of phobic stimuli to individuals affected by a specific phobia. Emphasis is given to the understanding of methodological variables that influence the acquisition and extinction of phobic reactions and their psychophysiological correlates, in order to provide methodological guidelines.

We take into account the different correlates of subliminal phobic stimulation, the physiological (such as skin conductance reactions, startle responses, heart rate variations, brain activations), the behavioral (avoidance of the phobic stimulus as assessed by the Behavioral Avoidance Test), and the subjective (such as the Spider/Snake Phobia Questionnaire and the Subjective Units of Distress Scale) ones. We aim at disentangling correlates that can be considered as the expression of a defensive survival circuit from those that can be considered as the expression of a cognitive circuit accounting for the conscious feeling of fear. Using this approach, we reconsider what fear is, how it is (dys)regulated in specific phobias, and we propose a therapeutic model based on a combination of subliminal and supraliminal desensitization procedures.

## Methods

### Study Design and Search Strategy

This systematic review has been created according to the Preferred Reporting Items for Systematic Reviews and Meta-Analyses (PRISMA) guidelines (Moher et al., 2009). PRISMA comprises a 27-item checklist to ensure and promote the quality of systematic reviews: this check-list is reported in Supplementary Table 1. The protocol employed in the current systematic review has been submitted for registration (ID number CRD42021129234) to the international prospective register for systematic reviews database (PROSPERO, https://www.crd.york.ac.uk/prospero/).

To develop an effective search strategy, we adopted the Population, Intervention, Comparison, Outcomes and Study Design (PICOS) worksheet (Moher et al., 2009). The PICOS strategy is summarized in Table 1.

**Table 1:** PICOS.

**Parameter**	**Inclusion criteria**	**Exclusion criteria**
Participants	Patients aged 18 or over, affected by specific animal phobia as assessed through questionnaires, behavioral tests, clinical interview; patients of any age reporting a significant level of specific fear as assessed through questionnaires, clinical interview and behavioral tests	Children; patients affected by non-specific phobia or by other disorders; enrolment of healthy subjects only; animal models
Interventions	Paradigms that guarantee unawareness of stimulus perception; covert paradigms checking for efficacy of subliminal stimulation; in case of masking, stimulus presentation shorter than 35 ms	Paradigms not guaranteeing unawareness of stimulus perception
Comparisons	Specific phobic vs. healthy participants; specific phobic participants in experimental group vs. specific phobic participants in control group; participants phobic for a specific animal/object vs. participants phobic for another animal/object	Healthy participants in experimental group vs. healthy participants in control group
Outcomes	Assessment of: phobia levels; subjective fear induced by exposure to phobic stimulus; behavioral measures of phobic avoidance; efficacy of covert stimulation; psychophysiological correlates linked to fear reaction (EEG, fMRI, HR, SCLs, EDA, etc.)	Any methodological issues related to collection of psychophysiological correlates
Study design	Within subjects, cross sectional, randomized controlled, longitudinal, pre-post	Case reports; commentary or reviews; methodological issues and lack of replicability; articles not published in a peer-reviewed journal; articles not available in full-text and/or in English language

The phase 1 was performed with a systematic search on the MEDLINE and SCOPUS electronic databases. An initial search was conducted in March 2018, and the final one was carried out in May 2020. Boolean operators were applied for combining a list of keywords related to subliminal paradigms and a list of keywords related to the emotional/phobic correlates; Table 2 summarizes the search steps performed for each database. Keywords describing subliminal paradigms were retrieved from the scientific literature (e.g., Taschereau-Dumouchel et al., 2018a) choosing both generic terms, such as “subliminal” or “unconscious,” and terms specifically referring to some techniques aimed at making a stimulus subliminal, such as “backward masking” or “continuous flash suppression”; keywords referring to emotional/phobic domain were derived from Cognitive Atlas concept terms related to phobia and basic emotions (https://www.cognitiveatlas.org/; Poldrack et al., 2011).

**Table 2:** Study research.

**Database**	**Steps**	**Query**	**Research in**	**Items found**
PubMed	#1	“backward masking” OR “backward masked” OR masking OR “attentional blink” OR subliminal OR “under threshold” OR under-threshold OR subthreshold OR sub-threshold OR “below threshold” OR “under perceptual threshold” OR “threat processing” OR covert OR “covert stimuli” OR “covert stimulus” OR “fear conditioning” OR “perceptual masking” OR preattentive OR pre-attentive OR unconscious OR “not conscious” OR “non conscious” OR priming OR implicit OR “continuous flash suppression” OR “flash suppression” OR subconscious	Title/Abstract	111,326
	#2	emotion OR emotions OR fear OR sadness OR disgust OR anger OR surprise OR trust OR distrust OR anticipation OR phobia OR threat OR electroencephalogram OR startle	Title/Abstract	267,576
	#3	Intersect #1 AND #2		10,894
	#4	Exclude reviews and case reports		9,689
	#5	Limit to “Humans”		4,006
	#6	Limit to “English” language		3,898
Scopus	#1	“backward masking” OR “backward masked” OR masking OR “attentional blink” OR subliminal OR “under threshold” OR under-threshold OR subthreshold OR sub-threshold OR “below threshold” OR “under perceptual threshold” OR “threat processing” OR covert OR “covert stimuli” OR “covert stimulus” OR “fear conditioning” OR “perceptual masking” OR preattentive OR pre-attentive OR unconscious OR “not conscious” OR “non conscious” OR priming OR implicit OR “continuous flash suppression” OR “flash suppression” OR subconscious	Title/Abstract	310,050
	#2	emotion OR emotions OR fear OR happiness OR joy OR sadness OR disgust OR anger OR surprise OR trust OR distrust OR anticipation OR phobia OR threat OR startle	Title/Abstract	812,921
	#3	Intersect #1 AND #2		17,570
	#4	Exclude reviews and case reports		16,382
	#5	Limit to “English” language		15,699

The selected articles resulting from the PUBMED and SCOPUS search were merged into a non-redundant database. Duplicates were removed using Mendeley desktop reference manager (http://www.mendeley.com).

## Results

### Flow Diagram

**Figure 1:**
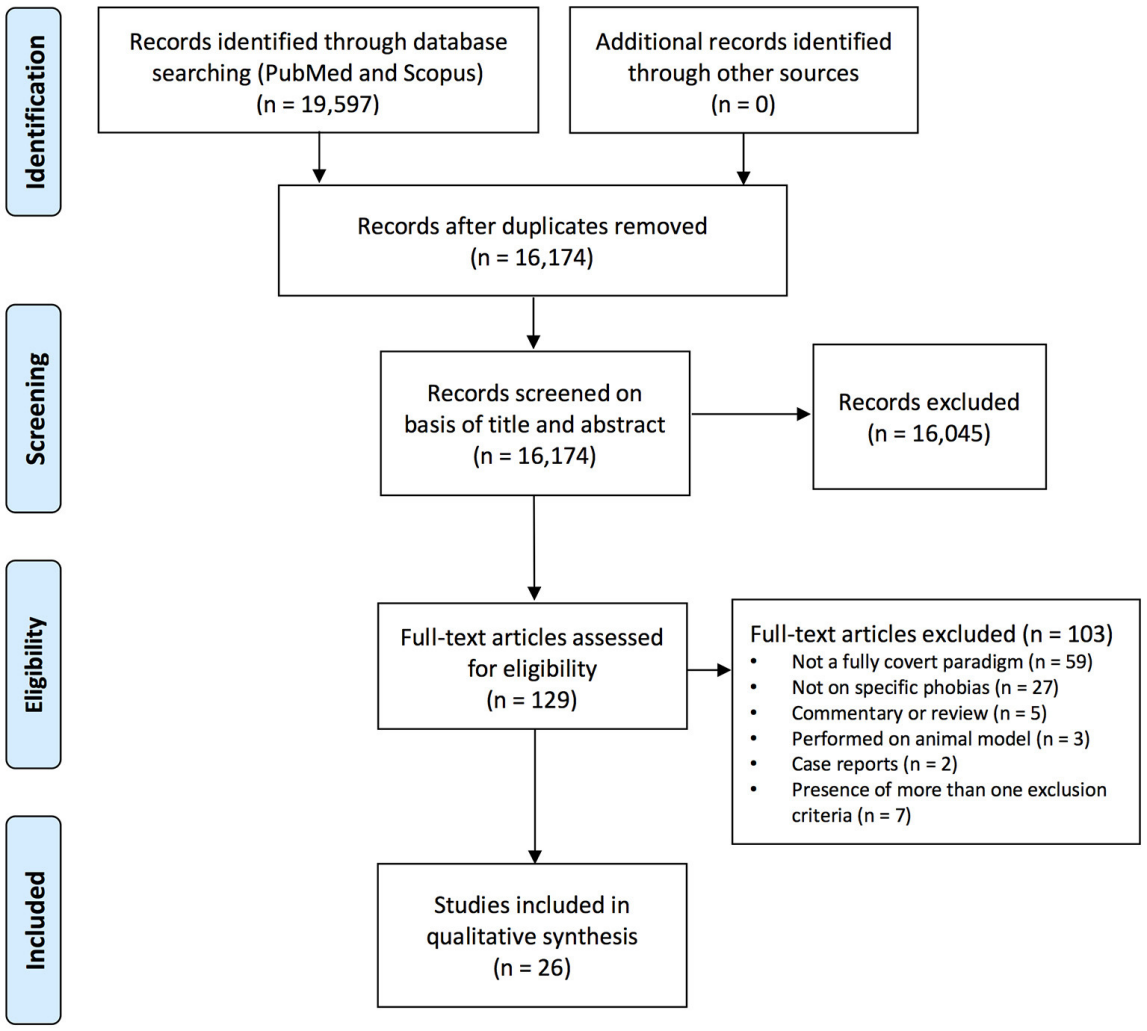
Flow diagram (selection process overview).

The retrieve process from scientific literature databases has been reported in Table 2. Items retrieved from PubMed and Scopus databases were merged in a non-redundant list containing 13,174 items. Since the database query was not restricted to specific keywords, but included common terms (e.g., fear; see Table 2), the pool of selected items was large. A further selection process, illustrated in Figure 1, was applied to screen the pool that was finally reduced to 26 studies.

### Study Selection and Characteristics

Three independent reviewers (SF, SG, and DC) checked the pool of 13,174 abstracts collected from PUBMED and SCOPUS search engine outputs (excluding duplicates): any disagreement was discussed with DM as arbiter. Titles and abstracts were screened, and 13,045 studies were removed because they met one or more exclusion criteria for the systematic review (Table 1). No existing reviews on the specific topic of interest (subliminal phobic stimulation in phobic subjects) were found. In most cases, stimuli were not fully subliminal, and/or were not phobic stimuli. The remaining 129 full-text papers were checked for the eligibility. At the end of this process, 26 articles meeting the eligibility criteria were identified.

Table 3 shows comparative information on study methods and outcomes of the 26 papers included in this review. Most of these studies demonstrated a significant effect elicited by the subliminal stimulation on the subjective, behavioral or physiological correlates. Mayer et al. (1999a) and Peira et al. (2012) designed experiments that involved different time durations in backward masking: different outcomes are showed for each variation.

**Table 3:** Retrieved studies and their main outcomes.

**References**	**Phobic animal**	**Assessment of specific phobia**	**No. phobics (no. males)**	**Mean age (DS)**	**Covert paradigm for phobic stimuli**	**Stimulus duration**	(^*^Significant) correlates of subliminal stimulation
Öhman and Soares (1994)	Spider	SFQ Spiders; SFQ Snakes	8 (M = 4)	27.1 (NA)	Backward masking	30 ms	^*^SCRs Self-assessment mannequin
Merckelbach et al. (1995)	Spider	SPQ; BAT; clinical interview	17 (M = 0)	32 (NA)	Backward masking	30 ms	Eyeblink startle reflex
van den Hout et al. (1997)	Spider	SPQ	37 (M = NA)	NA (NA)	Masked Stroop task (Backward masking)	20 ms	^*^Stroop interference
Thorpe and Salkovskis (1997)	Spider; various	Dimensions of spider phobia	34 (M = 4)	26.3 (NA)	Masked Stroop task (Backward masking)	16.6 ms	Stroop interference
Mayer et al. (1999a)	Spider	SPQ	47 (M = 0)	27.8 (NA)	Backward masking	15 ms 20 ms 30 ms	SCRs SCRs ^*^SCRs
van den Hout et al. (2000)	Spider	SPQ; FSQ	38 (M = 1)	32 (8.3)	Backward masking	20 ms	^*^SCLs
Wikström et al. (2004)	Snake	SPQ; clinical interview	19 (M = 0)	32,26 (9.31)	Masked Stroop task (Backward masking)	17 ms	SCLs Stroop interference
Carlsson et al. (2004)	Spider; snake	SPQ^a^; clinical interview	16 (M = 0)	26 (5)	Backward masking	14 ms	^*^Positron emission tomography (PET)
Carretié et al. (2005)	Spider	Dedicated questionnaire	31 (M = 8)	21.35 (2.32)	Various paradigms^b^	50 ms	^*^Electroencephalography (EEG)
Ruiz-Padial et al. (2005)	Spider	SPQ	18 (M = 0)	19.83 (1.85)	Backward masking	30 ms	^*^Heart rate response ^*^Cardiac defense response Dedicated questionnaires
Granado et al. (2007)	Spider	SCID; BAT; FSQ	25 (M = NA)	31.3 (7.4)	SpiderLess Arach- nophobia Therapy^c^	/	^*^BAT ^*^SUDs
Siegel and Weinberger (2009)	Spider	FSQ	40 (M = 9)	19.3 (2.3)	Very Brief Exposure (Backward masking)	25 ms	^*^BAT SUDs
Weinberger et al. (2011)	Spider	FSQ; BAT	23 (M = NA)	19.48 (2.49)	Very Brief Exposure (Backward masking)	20 ms	^*^BAT
Sebastiani et al. (2011)	Spider	SPQ	18 (M = 2)	22.92 (2.46)	Backward masking	20 ms	^*^SCLs Heart rate response
Siegel et al. (2011)	Spider	FSQ; BAT	36 (M = 6)	19.7 (NA)	Very Brief Exposure (Backward masking)	25 ms	^*^BAT SUDs
Lipka et al. (2011)	Spider	SPQ; clinical interview	18 (M = 0)	25.56 (5.26)	Backward masking	13 ms	^*^fMRI
Siegel and Weinberger (2012)	Spider	FSQ	101 (M = 25)	19.4 (1.8)	Very Brief Exposure (Backward masking)	25 ms	^*^BAT SUDs
Peira et al. (2012)	Spider	SAS for spiders	19 (M = 0)	25 (6.58)	Backward masking	10 ms 30 ms	EDA Heart rate Emotion ratings
Gutner et al. (2012)	Spider	BAT	24 (M = 3)	23.67 (8.56)	Backward masking	20 ms	^*^DS-R BAT SUDs
Siegel and Warren (2013)	Spider	FSQ; BAT	35 (M = 5)	19.7 (NA)	Very Brief Exposure (Backward masking)	25 ms	^*^BAT ^*^SUDs
Lipka et al. (2014)	Spider	SPQ; BAT; SBQ; affective ratings	14 (M = 0)	25 (3.7)	Backward masking	13 ms	^*^fMRI Symptoms reduction
Siegel and Gallagher (2015)	Spider	FSQ; BAT	86 (M = NA)	19.4 (NA)	Very Brief Exposure (Backward masking)	33 ms	^*^BAT SUDs
Schmack et al. (2016)	Spider	SPQ	25 (M = 8)	24.1 (0.7)	Continuous Flash Suppression (CSF)	/	^*^fMRI Affective ratings
Siegel et al. (2017)	Spider	FSQ; BAT; Spider Stroop^d^	21 (M = 0)	19.7 (1.6)	Very Brief Exposure (Backward masking)	33.4 ms	^*^fMRI Fear ratings
Siegel et al. (2018)	Spider	FSQ; BAT	60 (M = 10)	19.6 (1.5)	Very Brief Exposure (Backward masking)	33.4 ms	^*^BAT ^*^SUDs SCLs
Taschereau-Dumouchel et al. (2018a)	Various	Dedicated questionnaire	17 (M = NA)	NA (NA)	Decoded Neural Reinforcement^e^	/	^*^SCRs ^*^fMRI

*BAT, Behavioral Avoidance Test; DS-R, Disgust Scale – Revised; EDA, Electro Dermal Activity; fMRI, functional Magnetic Resonance Imaging; FSQ, Fear of Spiders Questionnaire; NA, not available data; SAS, Spider Anxiety Screening; SBQ, Spider Phobia Beliefs Questionnaire; SCID, Structured Clinical Interview for DSM; SCLs/SCRs, Skin Conductance Levels/Reactions; SPQ, Spider/Snake Phobia Questionnaire; SUDs, Subjective Units of Distress scale*.

*/, data missing because of the methodological properties of the experiment*.

* *Significant correlation with the phobic subliminal stimulus*.

a *Short versions of both Spider and Snake Phobia Questionnaire were used*.

b *Carretié et al. made the stimulus covert inducing inattentional blindness and showing it (a) briefly (50 ms), (b) in the periphery of the screen, (c) degraded through the partial overlap of a uniform black noise*.

c *Granado et al. masked the stimuli by selecting pictures that include arachniform perceptual features – even not representing a spider*.

d *Differently from the Masked Stroop task used in other papers, Spider Stroop showed visible spider-related words*.

e *In this paradigm, no stimulus was externally administered: an algorithm calculated the realtime activation of brain areas previously assessed to decode the categorization of phobic animal*.

### Synthesized Findings

Below, findings from the retrieved articles will be considered in relation to the following themes: heterogeneity in experimental paradigms, physiological correlates to subliminal phobic stimulus and assessment of subjective (i.e., conscious) fear caused by subliminal phobic stimulus presentation.

#### Experimental Paradigms

Several procedures allowed subliminal presentation of a phobic stimulus (see Table 3). Using backward masking (a method that makes stimuli subliminal by immediately covering them with different stimuli), Öhman and Soares (1994) first reported the induction of “unconscious anxiety” (based on a physiological index) in phobic participants. Subsequently, backward masking has become the most widespread method in this field: many papers included in the current systematic review employed this approach (see Table 3) or its variants. For example, Masked Stroop is a variation of Stroop Test that uses specific type of backward masking to assess the Stroop interference generated by subliminally presented primes. However, some authors have cautioned against the assumption that backwardly masked target stimuli are not consciously perceived and advocate the use of alternative terminology. Thus, Siegel and Weinberger (2009) employed a backward masking procedure, referring to it as “very brief exposure” of subsequently masked pictures. However, the duration of target stimulus presentation was similar to that used in other backward masking studies.

Besides backward masking, other approaches have been retrieved for making a phobic stimulus subliminal. Carretié et al. (2005) made stimuli subliminal by applying concurrent conditions. Stimuli had to be presented (a) briefly (50 ms), (b) in the periphery of the screen, (c) degraded with overlapping uniform impulsive noise, and (d) while inducing an inattentional blindness by asking participants to attend to a number in the center of the screen. In 2007, Granado et al. proposed an exposure protocol for treating spider phobia in which no images of spiders were shown (SLAT, SpiderLess Arachnophobia Therapy). Participants approached pictures previously judged as spider-like by non-phobic raters such as an office chair, or the Atomium sculpture in Bruxelles that share some specific perceptual features with spiders. When shown to spider phobics unable to undergo systematic overt desensitization, these images elicited no subjective distress and treated phobics exhibited a significant improvement over a 4-week period since they presented reduced subjective distress and avoidance in response to a caged tarantula.

Finally, a recent paper by Taschereau-Dumouchel et al. (2018a) deserves mention because the adopted paradigm did not involve any external stimulus. They proposed the Decoded Neural-Reinforcement technique, that automatically detects the activation of regions of interest in the brain and rewards it in real time: this method is supposed to shift the activation of the targeted regions from the original triggering stimulus to a new one, typically characterized by a different emotional valence. By doing so, Decoded Neural-Reinforcement technique can be considered a particular case of counter-conditioning, since it shifts to positive the negative valence associated to a stimulus by rewarding the activation of brain areas decoding for such stimulus. Applying Decoded Neural-Reinforcement technique to spider-fearful subjects, Taschereau-Dumouchel et al. (2018a) rewarded in real time the spontaneous brain activity observed in the specific regions previously identified in non-phobics being selectively activated by the phobic animal (an animal categorization task was performed in non-phobics). In phobic participants, counter-conditioning (the production of a response to a stimulus previously conditioned to produce a different response) induced by an economical rewarding of spontaneous activation of these regions was found to reduce subsequent physiological responses (skin conductance and amygdala hemodynamic activity) to images of feared animals.

Other paradigms that modulate awareness of phobic stimulus were excluded from this review because they did not satisfy the inclusion criteria requiring complete unawareness of the stimulus.

#### Correlates of Experiments Using Subliminal Paradigms

##### Self-Reported Measures of Fear and Disgust

All studies considered in this review used questionnaires measuring subjective fear for a specific animal, in order to assess phobia severity (see Table 3). The majority of studies enrolled participants with spider phobia, screened using questionnaires such as the Spider Phobia Questionnaire (SPQ; Klorman et al., 1974) and the Fear of Spiders Questionnaire (FSQ; Szymanski and O'Donohue, 1995).

Concerning the effect of subliminal stimulation on subjective fear, some studies used self-report measures, including the Subjective Units of Distress Scale (SUDs), the Behavioral Avoidance Test (BAT), the Disgust Scale – Revised (DS-R; Haidt et al., 1994, modified by Olatunji et al., 2007), the Self-Assessment Mannequin (SAM; Bradley and Lang, 1994) and dedicated measures of emotion (in Peira et al., 2012; Schmack et al., 2016; Siegel et al., 2017). It is worth noting that most of authors described BAT scores in terms of self-report measure of fear, although this is debatable.

Using backward masking, a reduction in spider avoidance (as assessed through BAT) was reported (Weinberger et al., 2011), but not in levels of subjective fear (as assessed through SUDs) (Siegel and Weinberger, 2009). A variant of backward masking (Very Brief Exposure) led to similar results: a reduced avoidance, but an unchanged fear (Siegel et al., 2011; Siegel and Weinberger, 2012).

Using backward masking with different target stimulus exposure times, no significant effect on emotion ratings was found by Peira et al. (2012): however, the authors also found that stimuli were subliminal in the 10 ms condition only.

Gutner et al. (2012) reported that subliminal stimulus exposure, combined with administration of *d*-cycloserine (a NMDA receptor agonist that enhances extinction learning of fear), produced a reduction in disgust levels (as assessed through DS-R) but not in fear levels (as assessed through BAT and SUDs).

Granado et al. (2007) reported a significant improvement in both the SUDs and BAT measures of phobic patients undergoing the SpiderLess Arachnophobia Therapy (SLAT).

A few studies have examined the association between self-report and objective measures in phobic participants exposed to subliminal phobic stimuli. Siegel et al. measured the effect of subliminal exposure on subjective fear of phobic subjects after previous supraliminal exposure (Siegel and Warren, 2013; Siegel et al., 2018). They found both a reduction in avoidance (as assessed through BAT) and in fear levels (as assessed through SUDs). Subliminal exposure did not induce higher fear, relative to supraliminal exposure (Siegel and Gallagher, 2015; Siegel et al., 2017): nevertheless, its beneficial effect on behavioral avoidance can last for at least 24 h (Siegel and Gallagher, 2015).

Schmack et al. (2016) assessed affective ratings for phobic pictures to check if their affective value could accelerate their access to awareness: this hypothesis was confirmed. Decoded Neural-Reinforcement was demonstrated to be effective in reducing psychophysiological variables, but not subjective fear (Taschereau-Dumouchel et al., 2018a).

##### Skin Conductance

Dermal sweat gland activity is controlled by the sympathetic nervous system and thus skin conductance provides an index of psychophysiological arousal (Boucsein et al., 2012).

Eight out of twenty-six studies investigated changes in skin conductance following exposure to phobic stimuli (see Table 3). Across articles, skin conductance measures include skin conductance level (SCL), skin conductance response (SCR), electrodermal activity (EDA) and galvanic skin response (GSR).

Only some studies reported significant skin responses. Öhman and Soares (1994) studied skin conductance while stimulating phobic participants through backward masking and reported changes in SCRs that were not accompanied by changes in conscious emotion. Subliminally-presented phobic words (e.g., “spider”) were reported to induce a significant Stroop effect (van den Hout et al., 1997) and skin conductance reactions (van den Hout et al., 2000). However, skin conductance reactions were not specific since similar changes were observed also in response to generic threatening words (e.g., “murder”), and the study lacked a control group (van den Hout et al., 2000). Other researchers found no effect in response to masked phobic words, in terms of Stroop interference (Thorpe and Salkovskis, 1997; Wikström et al., 2004) and skin conductance levels (Wikström et al., 2004). Finally, Sebastiani et al. (2011) reported skin conductance reactions significantly greater for masked shapes of spiders than for masked shapes of squirrels and crabs (the latter sharing with spiders similar physical features, but different emotional significance). Notably, skin conductance reactions were elicited as a function of emotional significance, regardless of physical similarity.

##### Startle Reaction

Based on its link with the thalamo-amygdala pathway, startle reflex was studied in one of the included papers (Merckelbach et al., 1995). The observation of eyeblink startle reflex in response to phobic pictures subliminally administered to phobic patients led to negative results: the only significant differences were reported in spider phobics showing a larger startle response in the second block of backwardly-masked phobic pictures, relative to controls (Merckelbach et al., 1995).

##### Cardiac Defense Reactions

Heart rate was measured only in 3 of the selected papers (see Table 3), to check if changes commonly reported in response to overt emotional stimuli are elicited by subliminal stimuli too. Ruiz-Padial et al. (2005) found no increase in heart rate following either subliminal or supraliminal phobic stimulation in spider phobics. However, the same stimuli did enhance the cardiac defense reaction to acoustic startle. Heart rate increased following the startle stimulus and the magnitude and duration of this response was increased following both supraliminal and subliminal exposure to spider images. In addition, subliminal spider images also increased the perceived unpleasantness of the acoustic stimulus. In spider phobics, Sebastiani et al. (2011) found no specific change in heart rate but increased skin conductance following subliminal phobic stimulation. In contrast, supraliminal spider images elicited both heart-rate acceleration and increased skin conductance. Neither supraliminal nor subliminal presentation evoked any response in non-phobic participants. Similarly, Peira et al. (2012) failed to detect significant increases in heart rate response to subliminal stimulus exposure.

Overall, studies did not detect significant increases in heart-rate of phobic participants exposed to subliminal phobic stimuli (Ruiz-Padial et al., 2005; Sebastiani et al., 2011; Peira et al., 2012). However, given the small number of studies, a conclusion cannot be drawn and methodological variables could moderate heart rate reactions to subliminal stimuli since both target-mask sequencing and number of trials have been reported to oppose heart rate acceleration in response to phobic stimuli (Ruiz-Padial et al., 2005).

##### Brain Activation Correlates

Seven studies have investigated the effects of subliminal phobic stimuli on brain activation by using functional magnetic resonance imaging (fMRI), electroencephalography (EEG) or positron emission tomography (PET). Table 4 summarizes brain areas specifically involved in subliminal exposure to phobic stimuli.

**Table 4:** Brain areas relevant for processing of phobic stimuli.

**References**	**Expo**	**Amygdala**		**Anterior insula**	**Fusiform gyrus**		**ACC**	**Prefrontal cortex**				
		**Left**	**Right**		**Left**	**Right**		**Ventromedial**	**Ventrolateral**	**Lateral**	**Dorsomedial**	**Dorsolateral**
Carlsson et al. (2004)	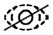	↑	↑	–			–					
	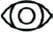	↑	↑	↑			↑					↓
Carretié et al. (2005)	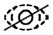											
	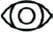							↑				
Lipka et al. (2011)	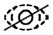	–	↑		↑	↑						
	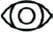	↑	↑		↑	↑						
Lipka et al. (2014) PRE-CBT	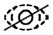	–	↑	–	–	↑	–	–			–	
	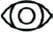	↑	↑	↑	↑	↑	↑	↑			↑	
Lipka et al. (2014) POST-CBT	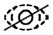	–	↑	–	–	↑	–	–			–	
	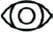	↑	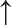 ^a^	↑	↑	↑	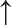 ^a^	↑			↑	
Schmack et al. (2016)	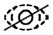	–	↑									
	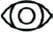	–	↑									
Siegel et al. (2017)	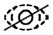	↑	↓	↑			↑	–	↑	↑	↑	↑
	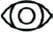	↓	–	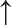 ^b^			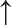 ^b^	↓	↓	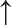 ^b^	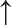 ^b^	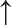 ^b^

*Most of studies investigating brain activity in response to subliminal and supraliminal exposition to phobic stimuli show a different involvement of right and left amygdala and of other cortical and subcortical structures. Siegel et al. (2017) report opposite results with respect to most of previous papers*.

*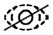, subliminal exposure; 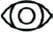, supraliminal exposure; ACC, anterior cingulate cortex; CBT, cognitive-behavioral therapy; –, not significant change; 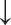, significant deactivation; 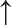, less significant activation; 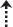, significant activation*.

a *In Lipka et al. (2014) the minor significance of region's activation, as revealed by group-by-time interaction analysis, show the effects of CBT on neural responses elicited by phobic pictures*.

b *In Siegel et al. (2017) the minor significance of region's activation is defined in terms of posterior probability of positively activated regions, ranging from 97.5 to 99.9% (less significant activations are those approaching 97.5% value)*.

PET scans during subliminal stimulation showed undifferentiated left amygdala responses for both phobic and fear-relevant (but non-phobic) stimuli; during supraliminal stimulation, both right and left amygdala were activated by phobic stimuli (together with anterior cingulate cortex, anterior insula, prefrontal cortex and periaqueductal gray) but not by fear-relevant stimuli (Carlsson et al., 2004). Furtherly, some prefrontal areas (dorsolateral prefrontal cortex and lateral orbitofrontal cortex) resulted deactivated by supraliminal phobic stimulation, relative to supraliminal fear-relevant stimulation.

EEG correlates of subliminal stimulation were investigated by Carretié et al. (2005). They used low-resolution brain electromagnetic topography (LORETA) and demonstrated a role of ventromedial prefrontal cortex (VMPFC) in directing attention to phobic stimuli and in facilitating their processing. The authors argue that VMPFC activation indicates a top-down control for the processing of subliminal stimuli (Carretié et al., 2005).

The other studies performed fMRI paradigms. Lipka et al. (2011) reported that the two amygdalae were differentially activated in supraliminal and subliminal processing of phobic stimuli. Both amygdalae showed a significant activation in supraliminal presentations, but the right amygdala also showed a specific activation in response to a subliminal stimulation. Interestingly, this preconscious activation of right amygdala positively correlated with interindividual variations in environmental threat monitoring. Additionally, significant activation of the fusiform gyrus, a structure implicated in processing facial emotional valence (e.g., Schmidt et al., 2020), was observed in both supraliminal and subliminal conditions (Lipka et al., 2011).

Subsequently, the same authors replicated and extended previous findings (Lipka et al., 2014): supraliminal stimuli exposure was associated with activation in several structures—amygdala, fusiform gyrus, insula, and the anterior cingulate and dorsomedial prefrontal cortices—and subliminal stimulus exposure produced activation of the amygdala and fusiform gyrus in the right hemisphere only. The same study examined also the effects of cognitive behavioral therapy (CBT) on specific phobia. Brain activations to supraliminal stimuli were reduced by CBT, most markedly in the right amygdala and anterior cingulate, but responses to subliminal stimuli were unaffected. For supraliminal stimuli, self-reported symptom improvement was significantly correlated with the reduction in amygdala responsiveness.

Schmack et al. (2016) used fMRI to identify the regions related to stimulus entry into conscious awareness. Activity in orbitofrontal and ventral visual areas was associated to conscious awareness of the stimuli, whereas subcortical activations, in regions such as amygdala, were associated to non-conscious stimulus processing.

Siegel et al. (2017) compared the effects of supraliminal and subliminal phobic stimulus presentation in phobic and non-phobic subjects. Images of spiders reduced the activity in default mode network more in phobics than in control subjects. This was claimed to reflect the greater salience of spider stimuli to phobic individuals. In phobic subjects, overt phobic stimulus presentation was also associated with deactivation of ventral prefrontal and temporal cortical regions implicated in emotion regulation and language processing, respectively. In phobic participants, subliminal phobic stimuli were associated with stronger activation of subcortical and cortical areas involved in language (Siegel et al., 2017). The authors interpreted these data as indicating weaker cortical regulation of regions involved in automatic stimulus processing.

Combining in real time fMRI scanning with counter-conditioning, Taschereau-Dumouchel et al. (2018a) attempted to produce desensitization-like outcomes without any exposure to the phobic stimulus. Their method (Decoded Neural-Reinforcement) automatically detects real-time activations of regions of interest in the brain and reinforces it through economical reward. The noticeable result was the successful counter-conditioning of some physiological correlates of fear response: skin conductance and amygdala activity decreased for counter-conditioned phobic stimuli but not for a second phobic stimulus that was not counter-conditioned (Taschereau-Dumouchel et al., 2018a).

## Discussion

The systematic review on studies that apply subliminal phobic stimulations to phobic subjects allowed the retrieving of 26 papers. Papers used different subliminal paradigms and investigated different correlates.

The overall prevalence of specific phobia is estimated at ~7% in the general population, with a higher prevalence of women (Eaton et al., 2018). Also, age differences in prevalence have been reported, however the range differs depending on the feared object or situation (Fredrikson et al., 1996). Overall, variations in prevalence have been explained by evolutionary forces: greater sensitivity to danger in females of child-rearing age may reflect an adaptive trait (Eaton et al., 2018). Selected studies reflect this distribution: some studies enrolled women exclusively and even when both sexes were present, usually, more females than males were recruited (see Table 3). Sex differences in psychophysiological reactions to emotional stimuli have been reported: phobic women display greater sensitivity to threat signals as compared to men (Cahill et al., 2001; Williams et al., 2005). This suggests the probability of positive findings will be influenced by sample sex ratio. Moreover, the number of phobic subjects varied from 8 (in the seminal paper by Öhman and Soares, 1994) to 86 (Siegel and Gallagher, 2015), with a median of 25 subjects and studies recruited predominantly young people (see Table 3), typically University students. Thus, also the differences in sample size and composition between studies may explain some inconsistencies in the literature.

Several methods were used to screen for the specificity and severity of specific phobia in participants (see Table 3). In most cases a self-report questionnaire (typically, the SPQ or FSQ) was employed sometimes in combination with the behavioral avoidance test (BAT). Only a few studies administered a clinical interview (see Table 3). Subjects were considered phobic if, at a minimum, they scored in the top 25% of fear ratings assessed by the questionnaire (e.g., Sebastiani et al., 2011), and were assigned to the control group if they were at least in the bottom 50% (e.g., Siegel et al., 2011). How well such an approach distinguishes clinical from non-clinical cases may be an issue as the cut-off score defining the top quartile will be determined by the distribution of scores for the whole sample. This complicates the comparison of results between studies since relevant differences in phobia severity may be hidden. Appropriate reporting of test scores for both the overall sample and experimental subgroups should be adopted as a minimal standard. Thorough investigation of the psychometric properties of all screening tools, including specificity and selectivity measurements, and the identification of standardized cut-off scores (e.g., Ovanessian et al., 2019) would also facilitate future research.

All studies had to cope with the issue of assessing awareness for masked stimuli (Wiens, 2006). The retrieved research studies using masking-based subliminal stimulation paradigms (22/26 publications) indicate that both stimulus awareness and elicitation of psychophysiological reactions are related to stimulus duration, however reported exposure times varied from 10 ms (Peira et al., 2012) to 33.4 ms (Siegel et al., 2017, 2018), with a mean of 22.6 ms. Significantly, some authors found evidence of awareness when masked stimuli were presented for more than 15 ms (Mayer et al., 1999a), whereas others have reported unawareness using longer durations (e.g., 30 ms in Öhman and Soares, 1994; 33,4 ms in Siegel et al., 2018). Previous reports indicate that awareness thresholds vary widely between individuals and that these differences may derive from a variety of sources (Maxwell and Davidson, 2004; Pessoa et al., 2005; Edwards et al., 2006). Such evidence highlights that stimulus exposure duration *per se* cannot be used to reliably define the limits of conscious awareness/unawareness, suggesting that objective confirmation of stimulus (un)awareness is necessary.

### Psychophysiological Reactions

Psychophysiological reactions to threat have been widely studied in healthy subjects by means of overt stimulation paradigms and they typically relate to sympathetic activation as observable on skin conductance, heart rate and startle responses. Similar responses are reliably enhanced in phobic subjects exposed to overt phobic stimuli (Globisch et al., 1999). The retrieved papers measuring the same correlates in response to subliminal phobic stimuli reported contrasting results (see Table 3). In the following paragraphs, we comment the more debated evidence.

#### Is Skin Conductance Activity a Reliable Marker of Phobic Subliminal Stimulation?

Based on their observation that subliminal phobic stimuli can elicit skin conductance reactions in phobic participants, Öhman and Soares (1994) hypothesized that phobias are hard-wired fears driven by an innate and unconscious detection system that recognizes specific objects or animals thanks to their visual features. These authors speculated that phobias might involve activation of subcortical neural circuits responsible for rapid processing of the gross features of emotional stimuli (LeDoux, 1994). Subsequent research, much of which has been conducted in non-phobic subjects, continues to support this view, and stimulated further investigation on the consequences of subliminal exposure to phobic stimuli. However, contrasting results emerged from the present literature review concerning skin conductance responses to subliminal phobic stimuli.

**Figure 2:**
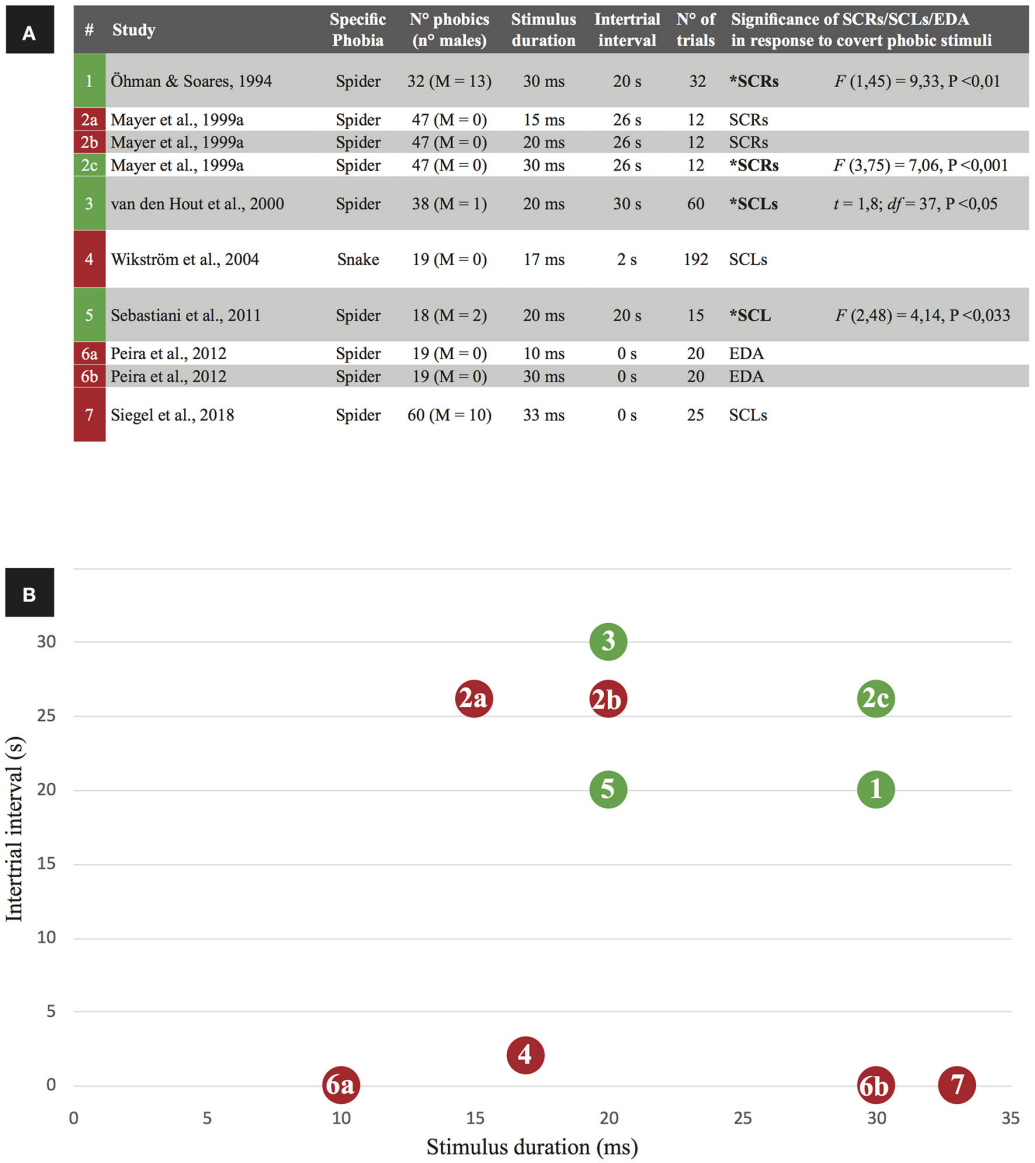
Skin conductance as a correlate of phobic subliminal stimulation in the retrieved studies. **(A)** Green box indicates a study with positive results (significant increases of skin conductance levels are reported), a red box indicates negative results. For each paper, exposure properties of the phobic stimulus (duration, intertrial interval, number of trials) are listed: based on stimulus duration and intertrial interval, **(B)** visually represents the relation between those variables in predicting the significance of skin conductance activation.

Four studies reported positive results (increased skin conductance following subliminal stimulus exposure) while seven studies reported negative results (Figure 2A). On balance, negative findings were more common, suggesting subliminal stimuli do not reliably increase skin conductance. However, Öhman (1999) has highlighted methodological issues in papers that claim no evidence for increased skin conductance response to subliminal phobic exposure, but others offered counter-criticisms (Mayer et al., 1999b). From a closer inspection of selected studies, intertrial interval (ITI; that is the time interval between trial offset and onset of the succeeding trial) and stimulus duration exposure are interacting determinants of skin conductance reactions to subliminal phobic stimuli (Figure 2). When studies employed larger intertrial interval (20–30 s) and longer exposure to the phobic stimulus (not exceeding the unawareness threshold of 33 ms) they obtained successful elicitation of skin conductance in response to phobic subliminal stimulation. By contrast, studies employing shorter intertrial intervals and/or shorter stimulus exposure durations were associated to no change in skin conductance. A single exception violated this rule: Mayer et al. (1999a; #2b in Figure 2) reported unaltered skin conductance despite using long intertrial interval (26 s) and stimulus duration (20 ms). Actually, they observed significant increase in skin conductance only when employed a longer stimulus duration (30 ms). However, participants reported some degree of stimulus awareness at both the 20 and 30 ms durations, contrarily to a relevant body of evidence reporting unawareness for stimuli lasting even more (see Table 3). To account for such incongruence, authors highlighted that stimuli were presented in a non-random order.

The relationship shown in Figure 2 is coherent with the observation that intertrial interval has a significant effect on recovery and amplitude of electrodermal reactions (Boucsein et al., 2012). In particular, an intertrial interval of about 30 s—the same used in the papers successfully reporting a skin conductance reaction to phobic subliminal stimuli—is the recommended one for a proper electrodermal recovery (Breault and Ducharme, 1993). Accordingly, smaller skin conductance reactions to subliminal phobic stimuli will occur when shorter (<30 s) intertrial intervals are employed, as confirmed by negative results reported in Figure 2. A meta-analysis would provide the best evidence of the role of intertrial intervals and stimulus duration as co-determinants of skin conductance reactions to subliminal phobic stimuli; however, most of the selected papers failed to provide sufficient skin conductance data to support such quantitative analysis.

Other methodological differences that could account for contrasting results were considered (e.g., number of trials; task schedule), but no effect emerged. For example, task schedule (fixed or variable ratio, fixed or variable interval) is known to affect timing and efficacy of conditioning and extinction procedures, and could eventually affect the results listed in Figure 2 (if we interpret negative results in skin conductance as a signal of extinction). Nevertheless, such methodological differences were equally distributed between experiments reporting positive results for skin conductance reactions to subliminal phobic stimuli. In detail, two papers (Öhman and Soares, 1994; van den Hout et al., 2000) used a variable interval to separate stimuli (from 25 to 35 s), but the other two papers reporting positive results (2c condition in Mayer et al., 1999a; Sebastiani et al., 2011) used a fixed interval (respectively, 26 and 20 s). All experiments reported in Table 2 used a fixed ratio: task schedule, as well as the number of trials, does not account for positive or negative results. Importantly, one paper (Mayer et al., 1999a) reported results from three experiments adopting the same (fixed) interval but a different duration of stimuli: positive results were reported for the condition showing the longer-lasting stimuli, regardless of task schedule. This all considering, we can't exclude that some other methodological details could affect the results reported in the selected papers, but stimulus duration and intertrial interval seem to be the most influent factors to take in account when building experimental protocols aimed to investigate skin conductance reactions to subliminal phobic stimuli. These considerations should be particularly important for experimental protocols aiming to subliminally desensitize phobic patients (e.g., Siegel et al., 2018) and/or to counter-condition their reaction to phobic stimuli (e.g., Taschereau-Dumouchel et al., 2018a).

#### Do Phobic Subliminal Stimuli Affect Cardiac Defense Reactions?

Psychophysiological research has long recognized that specific cardiac defense reactions occur in the presence of potential injury or death (Berntson et al., 1998). Early work identified that threats can trigger both decelerations and accelerations of heart rate, respectively, in response to moderate threat (or novelty) and intense threat. Heart rate deceleration is associated with increased stimulus processing (increased attention toward and perception of the stimulus) and passive coping behaviors (decreased environmental responsiveness, quiescence, immobility). Heart rate acceleration—in particular, following an initial deceleration—is also associated with a defense response to phobic pictures: however, coherently with the avoidance behaviors that characterize phobic patients, it results in a motivated inattention for the phobic stimuli (Ruiz-Padial et al., 2011).

Two out of three papers assessing cardiac correlates of subliminal phobic exposure (see Table 3) reported negative results (Sebastiani et al., 2011; Peira et al., 2012). By contrast, Ruiz-Padial et al. (2005) found a significant priming effect in cardiac defense response in subjects exposed to subliminal phobic stimuli. These data suggest that heart rate is minimally altered by subliminal phobic stimuli but that such stimuli could modulate cardiac defense reactions elicited by a startle probe. This conclusion is necessarily tentative, being based on 3 studies only. However, motivational modulation of acoustic startle is considered a highly sensitive method for probing the affective state of both human and non-human subjects (Lang, 1995). Furthermore, startle reflex potentiation has been reported in phobic subjects following overt phobic provocation (Hamm et al., 1997). Indeed, Ruiz-Padial et al. (2005) highlighted that their results supported Öhman's model of pre-attentive processing of fear. Further investigation of startle potentiation by subliminal phobic stimuli would be a valuable avenue for future research.

#### How Does Brain Activity Change in Response to Subliminal (Compared to Supraliminal) Phobic Stimuli?

EEG, PET, and fMRI techniques have been used to study central correlates of subliminal phobic stimulus processing (see Table 3) and results have been interpreted with respect to theories that presume the automaticity of emotions (e.g., LeDoux, 1994; Öhman and Soares, 1994). These techniques provide correlative rather than causal results and therefore do not provide definitive evidence regarding the validity of these theories.

LeDoux's theory of distinct pathways activated by evolutionary-salient stimuli (LeDoux, 1994) could account for—and find empirical confirmation in—results reported for subliminal and supraliminal exposure to phobic stimuli (Öhman and Soares, 1994). A consequent research hypothesis was that subliminal phobic stimuli should have been processed by subcortical structures mainly, whereas supraliminal stimuli should have been processed by wider areas of the brain, including cortices (Siegel et al., 2017). For example, amygdala activity drew attention due to its role in psychophysiological responses to aversive stimuli, including correlates observed in response to overt phobic stimuli (Globisch et al., 1999). Models based on non-phobic participants exposed to subliminal aversive stimuli (e.g., Gläscher and Adolphs, 2003) propose that the right amygdala is involved in fast, automatic processing of emotional stimuli and the left amygdala is involved in slower, more sustained and precise stimulus processing.

Those models may also apply for the results reported in phobic patients by Lipka et al. (2011), who found a specific response of right amygdala to subliminal phobic stimulation, which positively correlated with environmental threat monitoring. This finding was interpreted as supporting the idea that subcortical circuitry centered on the amygdala subserves the hypervigilance trait of phobic patients.

Evidence from several retrieved studies highlights that non-conscious processing of phobic stimuli involves both subcortical and cortical brain areas (see Table 4) and cautions against overly-simplistic interpretation that subcortical activations necessarily reflect unconscious/emotional processing and cortical activations necessarily reflect conscious/cognitive processing (Pessoa, 2008).

Another assumption made in literature (Siegel et al., 2017) is that supraliminal exposure should result in stronger neural activity than subliminal exposure. Although apparently reasonable, it results to be incorrect, at least with respect to phobic individuals. Siegel et al. (2017) reported increased changes in fMRI brain activations of phobic subjects in response to subliminal, rather than supraliminal, phobic stimuli. Similarly, a deactivation of areas involved in top-down regulation of emotions (dorsolateral prefrontal cortex and lateral orbitofrontal cortex) was reported for supraliminal phobic stimulation, relative to supraliminal fear-relevant (but non-phobic) stimulation (Carlsson et al., 2004).

In addition, some areas are specifically activated in either supraliminal or subliminal stimulations, and only few cortical areas are reported to be predictive of the stimulus access to awareness (see Table 4): Schmack et al. (2016) identified the left orbitofrontal cortex and the right fusiform gyrus. These results suggest a role of these structures in integrating information for its access to consciousness (Schmack et al., 2016).

Some studies have investigated brain activity responses to subliminal and supraliminal stimuli as a function of desensitization therapies. Patients who underwent CBT (incorporating supraliminal exposure to phobic stimuli) reported significant symptom reduction and showed reduced brain activations to supraliminal but not to subliminal phobic stimuli (Lipka et al., 2014). Patients who underwent the counter-conditioning procedure proposed by Taschereau-Dumouchel et al. (2018a) successfully eliminated skin conductance and amygdala activation responses associated with phobic stimuli without any exposure to those stimuli, however they did not investigate whether subjects reduced consciously experienced fear and risk of relapses.

The lack of a decrease in brain responses to subliminal phobic stimuli following CBT suggests that this approach does not impact on the regulation of automatic attentional processes (Lipka et al., 2014) and could explain the significant vulnerability to relapse that persists following successful elimination of fear via CBT (LeDoux, 1994; Vervliet et al., 2013). Actually, subliminal stimulations have a role in activating (and properly habituating) non-conscious stimulus-processing mechanisms and in fact recent evidence supports the idea that subliminal stimulus exposure may be an effective means of reducing relapse (Oyarzún et al., 2019). As a summary, there are hints that responses to subliminal stimuli survive standard interventions involving overt stimuli exposure only, and paradigms integrating also subliminal stimuli exposure might be more effective.

#### How Much Self-Report Levels of Fear Couple With Behavioral and Physiological Responses to Subliminal Phobic Stimulation?

Some authors have claimed that exposure to subliminal phobic stimuli can reduce fear to a comparable degree as supraliminal desensitization protocols determine (Siegel et al., 2011; Siegel and Weinberger, 2012; Siegel and Warren, 2013; Siegel and Gallagher, 2015).

Most retrieved articles assessed subjective fear by means of self-report questionnaires (see Table 3) and with the Behavioral Assessment Test (BAT). The usage of the BAT for assessing subjective fear was under the assumption that it would be associated with reduced avoidance (e.g., Siegel and Gallagher, 2015). However, behavioral measures were often proved to be unrelated to subjective fear (Siegel and Weinberger, 2009, 2012; Siegel et al., 2011; Gutner et al., 2012; Siegel and Gallagher, 2015; see Table 3). Thus, a reduced avoidance from phobic stimulus (as assessed with BAT) may occur in absence of felt fear reduction as declared in the self-report.

In fact, a weak coupling among subjective, behavioral and physiological components of fear (as well as other emotions) is not uncommon (e.g., Mauss et al., 2005; LeDoux, 2015) and can be affected by several factors: for example, a tighter coupling is more likely observed during intense emotions (Mauss et al., 2005).

Some of the retrieved studies indicate protocols for increasing the coupling between these dimensions. In order to observe decreased fear in terms of SUDs, phobic patients have to undergo a two-step procedure: (1) subliminal exposure; (2) single-stimulus supraliminal exposure (Siegel and Warren, 2013; Siegel and Gallagher, 2015). This effect was interpreted as the result of a cognitive remodeling that only occurs after a conscious appraisal of the phobic stimulus: consciously facing a phobic stimulus after a subliminal exposure would allow phobic patients acquiring, even subliminally, the decrease of physiological responses, and coding them as a reduction in subjective fear. At present, it still needs to be investigated whether the awareness degree of such reduction in physiological feedbacks correlates with a reduction of subjective fear felt when consciously facing the phobic stimulus.

The apparent incoherence between self-report and the behavioral and physiological measures of fear hinders the identification of the optimal way to measure it. We should consider fear for a given stimulus as the cognitive appraisal of physiological and behavioral feedbacks related to that stimulus, combined with own memories and beliefs. Self-report measures alone does not provide a full characterization of the fear construct, as memories and beliefs related to the phobic stimulus need a longer time to be cognitively remodeled; behavioral measures can be successfully modulated by subliminal extinction protocols, but patients will still feel the same level of fear for the phobic stimulus; and physiological measures show extinction phenomena without awareness.

A weighted combination of self-report, behavioral and physiological measures would result in a better long-term assessment of fear levels. Since quality and quantity of self-perceived psychophysiological reactions to phobic stimuli (subliminal and consciously perceived) subjectively reported by patients could affect onset and maintenance of phobic fear, it could be used also to reduce it in protocols involving a subliminal exposure. To this regard, in the Conclusion section we propose a model for addressing different correlates of fear elicited by subliminal exposure to phobic stimuli and for quantifying their contribution to the final subjective level of the consciously experienced fear.

### Proposals for Methodological and Terminological Standards

Based on papers examined in the review, we propose the following theoretical agreements and methodological standards for designing experimental protocols that involve subliminal phobic stimuli:

- the concept of phobic reaction must not be overlapped to the concept of subjective fear (LeDoux, 2014). Overlapping may cause a terminological ambiguity that also brought to hypothesize that a decrease in psychophysiological correlates of a phobic reaction necessarily corresponds to a parallel decrease in subjective fear. This parallelism is being criticized since decades (e.g., Lang et al., 2000), but it is still manifest in a terminological ambiguity. We embrace the terminology proposed by LeDoux (2014), distinguishing “fear”—intended as the subjective emotion felt—from the psychophysiological measures that typically correlate with fear. Thus, a measure of psychophysiological correlates or behaviors does not represent a satisfying measure of fear, as suggested since the pioneering works of Lang et al. that distinguished between valence-dependent (e.g., heart rate) and arousal-dependent (e.g., skin conductance) correlates (Lang et al., 1993, 2000). In the Conclusions section, we propose that the different correlates (subjective, behavioral, and physiological) of fear taken together result in a multidimensional measure that will better predict long-term levels of the consciously experienced emotion;
- consider that awareness threshold varies as a function of the salience that each stimulus has for each subject (Lang et al., 1993) and different thresholds depend on the clinical condition (e.g., phobic participant have lower awareness thresholds for phobic pictures; D'Alessandro et al., 2009; Schmack et al., 2016). Researchers administering subliminal phobic stimuli should just focus on inducing correlates of emotional responses in absence of experienced fear. In order to assess the absence of perceived fear in a subliminal extinction protocol, the experimenters should submit forced-choice questions concerning the emotional valence of each stimulus. This approach is similar to what is recommended for assessing the efficacy of masking (Wiens, 2006, 2007);
- do not assume a superposition effect in which psychophysiological correlates of supraliminal fearful stimuli correspond to the correlates of subliminal fearful stimuli, eventually magnified, together with some other specific correlates. Indeed, studies have highlighted brain activations greater for the subliminal stimulations compared to the supraliminal ones (e.g., Siegel et al., 2017);
- paradigms that make the phobic stimulus subliminal through a manipulation of exposure time (e.g., backward masking) must consider effects of methodological parameters on the psychophysiological correlates (see section Psychophysiological Reactions). We found that a stimulus duration smaller than 20 ms and an intertrial stimulus interval smaller than 20 s can hardly lead to significant skin conductance responses, probably due to the physiological features of such correlate. Finally, also the number of trials should be considered when designing the experimental protocol as suggested by results concerning habituation effect (e.g., Lipka et al., 2014);
- the concept of physiological extinction or habituation for a subliminal stimulus must not be overlapped with the concept of desensitization for the same stimulus when it is consciously perceived. Even if skin conductance correlated to subliminally administered phobic stimuli undergoes habituation, the phobic patient will still be afraid of the same phobic stimuli when consciously perceived. In terms of clinical outcomes, the habituation to subliminal stimuli is meaningless to the phobic patient, if he/she is still afraid of the supraliminal stimulus.

### Toward an Integrated Defensive-Cognitive Therapeutic Model

Only a minority of phobic patients undergo treatment, since it typically involves a supraliminal exposure to phobic stimuli (Stinson et al., 2007): among them, a relevant percentage is unresponsive to treatment (Loerinc et al., 2015) and occasionally presents with relapses (Eaton et al., 2018). This evidence could reflect a deficit in the inhibitory learning mechanisms needed to remodel patient's beliefs about the phobic stimulus: these theoretical implications were recently integrated in a therapeutic approach aimed to maximize the effect of exposure therapies (Craske et al., 2014). One of the key features of this approach is to make the patient more aware of some cognitive steps needed for a successful exposure therapy: for example, patients are explicitly requested to realize what they learnt thanks to their reaction to stimulus exposure. Similarly, we propose an approach aimed at overcoming some limitations that are possibly preventing subliminal exposure protocols from achieving satisfactory results in terms of therapeutic outcomes.

**Figure 3:**
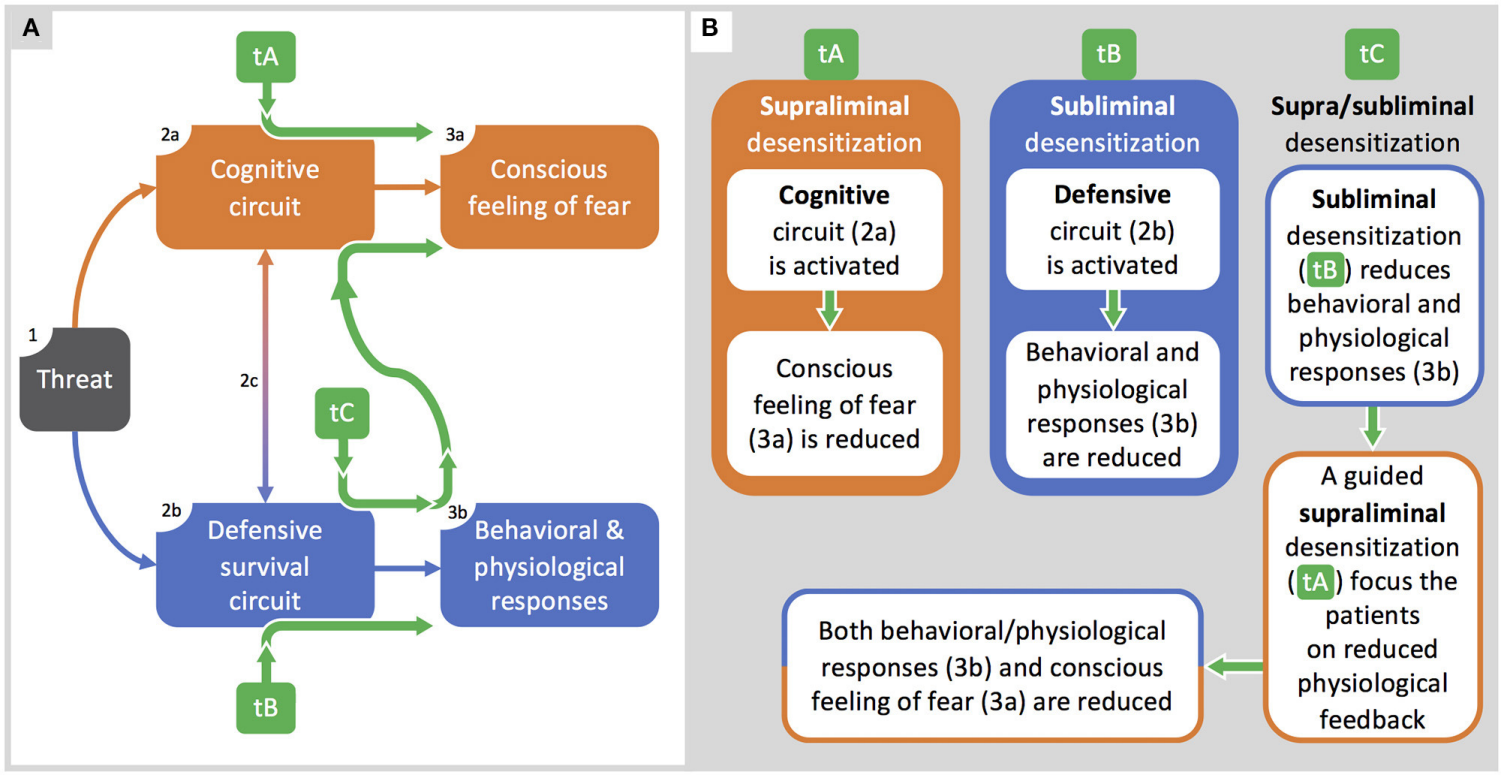
Integrated supra/subliminal therapeutic model. **(A)**: the model by LeDoux and Pine (2016) is integrated with the therapeutic pathways (green arrows) described in **(B)**. **(B)**: along with the representation of therapeutic protocols based on supraliminal (tA) or subliminal (tB) desensitization, here we propose a model (tC) integrating both tA and tB organized to optimize therapeutic outcomes.

The need for such an approach is based on the observation that patients treated with subliminal exposure to phobic stimuli, that mostly affect physiological correlates, still experience a subjective distress when consciously face the stimuli, as indicated by results from studies with BAT improvements in absence of any change in SUDs (Table 3). On this basis, subliminal and supraliminal processing behave as distinct factors, and their superposition misleads therapists and researchers to consider them as a single factor (Figure 3). In contrast, if phobias involve two distinct circuits—even if interacting with each other—they should be both adequately treated to reach the best efficacy.

Therefore, the effect of subliminal phobic stimulation seems to depend on the neural systems activated. If specific phobias arise because of an overreacting innate system that processes fearful stimuli (Seligman, 1971), habituation to subliminal phobic stimuli should also lead to a resolution of the conscious fear. On the other hand, if specific phobias arise because of because of conscious exaggeration of thoughts toward the phobic stimulus—eventually caused by traumatic experiences—(Mineka and Zinbarg, 2006), subliminal exposure is supposed to be totally ineffective.

These models do not necessarily contrast each other. An innate detection system automatically and unconsciously focuses attention for evolutionary-relevant stimuli such as spiders and snakes (Öhman and Soares, 1994). Coherently with the metaphor of emotions as soups resulting from many ingredients (LeDoux, 1994), this orientation response is initially unemotional, but it eventually represents the precursor of the emotional response when the stimulus catching attention is associated with a fearful event. Such an association would be the basis to develop specific phobia, however it must be consolidated by avoidance and overthinking behaviors that in turn would increase the arousal at the basis of orientation. The psychophysiological correlates of the subliminal phobic exposure could be mainly related to the initial orientation response since their elicitation and their extinction are unrelated to subjective fear in most of cases (see Table 3).

Different theories stand on the idea that two parallel processes are involved in onset and maintenance of specific phobias. In Mowrer's *bifactorial theory* (Mowrer, 1947, 1956), the phobic patient can be unaware of the event triggering the specific phobia through classical conditioning, but the phobia will be maintained through operant conditioning (intended, in that case, as an active and conscious avoidance of phobic stimulus). The *opponent-process* theory by Solomon (1980) applied to emotional regulation conjectures a slower, longer-lasting relief process that compensates a stronger, faster, scaring process for the phobic stimulus (Solomon and Corbit, 1974). The two processes proposed by Solomon share the same onset, but the slower one has an opposite valence balancing that of the faster process in the long term. LeDoux and Pine (2016) theorized the existence of two systems that respond to threatening stimuli, one accounting for behavioral and physiological responses and the other accounting for conscious feeling states as assessed by self-report measures. Similarly, Taschereau-Dumouchel et al. (2018b) adopted some ideas from higher-order theories of consciousness to propose a low-order circuit providing for defense (based on subliminal processes) and a high-order circuit providing for the conscious experiencing of fear (based on supraliminal processes). The innovative use of neurofeedback as a form of subliminal extinction (Taschereau-Dumouchel et al., 2018a) is fascinating because it suggests that a treatment of specific phobias without exposure to the phobic stimulus might be possible.

The interaction between these processes explains the emerging observation that subjective fear (typically measured by SUDs) seems to be affected by subliminal exposure only when its assessment follows also a conscious exposition to phobic stimulus (Siegel and Warren, 2013; Siegel and Gallagher, 2015). This phenomenon fits with strategies proved to maximize the effects of exposure therapy (Craske et al., 2014): for example, subliminal exposure to phobic stimuli could extinguish physiological correlates, but such extinction could have no recognizable effects until its coupling with the subjective feeling of fear induced by conscious exposure to phobic stimuli. Synthesized results from current systematic review support the aforementioned theories and are the basis for improving current treatments for specific phobias.

In Figure 3A, we use the LeDoux and Pine model (2016) to explain strengths and weaknesses of current therapeutic approaches and to propose a novel integrated one (Figure 3B). Thus, we compare classic therapeutic protocols based on exposure to visible stimuli (tA), recent protocols (e.g., Siegel and Warren, 2013) based on subliminal stimulations (tB), and our proposal of a therapeutic protocol based on both supraliminal and subliminal stimulation (tC). In Figure 3A, from left to right, when sensory systems detect a threatening stimulus (box 1), two circuits are activated in parallel: a cognitive circuit (box 2a) accounting for the conscious feeling of fear (box 3a), and a defensive survival circuit (box 2b) accounting for behavioral and physiological responses (box 3b). The two circuits have also reciprocal communications (arrow 2c) accounting for the unitary-experienced fear.

In Figure 3B, classical therapeutic protocols (tA) are effective in inducing desensitization for supraliminal phobic stimuli and in reducing the conscious feeling of fear, but they are hardly accepted by the most severe patients. Classical protocols can eventually lead to relapses or to a new phobia, if the malfunctioning defensive survival circuit was just rendered dormant rather than erased (LeDoux, 1994). Conversely, therapeutic protocols based on subliminal extinction (tB) can successfully reduce behavioral and physiological responses to phobic stimuli (box 3b): subliminal exposure is acceptable for the phobic patients but, by itself, it does not affect the conscious fear experienced when facing a phobic stimulus.

Based on the interactions between circuits, we propose the integrated therapeutic model (tC). As a first step, a subliminal exposure reduces physiological and behavioral responses to unconsciously perceived phobic stimuli: at this stage, it represents a latent desensitization. As a second step, patients are guided to become aware of the reduced physiological feedbacks when facing an overt phobic stimulus, this allowing a cognitive remodeling that will result in an enhanced reduction of fear. Such a two-step protocol will likely benefit from the strengths of both subliminal (tB) and supraliminal (tA) exposure, hopefully resulting in a more acceptable and effective treatment.

## Limitations

The present systematic review adheres to standardized protocols for selecting and evaluating papers to include, as reported in Methods section and meticulously detailed in Table 1, Supplementary Tables 2, 3. This strictness represents a relevant strength of this paper, allowing it to focus on comparable results and to avoid confusing outcomes that would had only increased the quantity of information, at the expense of quality: nonetheless, the present paper presents some limitations that must be acknowledged.

The strict inclusion criteria adopted resulted in the exclusion of a huge number of papers that didn't clearly measure the effectiveness of subliminal paradigm used to administer the phobic stimuli. This was made necessary by the lax interpretation that scientific literature used to give to the concept of subliminal stimulation, that led to methodological and terminological confusion (Wiens, 2006; LeDoux, 2014) and misleading descriptions of the results (LeDoux, 2014). On the other hand, the strictness of inclusion criteria is also reflected in a relatively small number of included studies (26; see Table 3): among those, the papers investigating therapeutic effects of subliminal exposure to phobic stimuli are 8 (see Table 3), most of which published by the same scientific group (Siegel et al.). Despite the strict criteria adopted, the included studies still show slight differences concerning the recruitment of phobic participants, the nature of phobic stimuli administered, and the paradigm used to make these stimuli subliminal.

All these limitations considered, the conclusions drawn by the present systematic review need to be confirmed by further studies. In particular, the integrated model proposed should rely on direct empirical demonstration that will hopefully be provided in future experiments.

## Conclusions

The aim of this review was to address scientific literature studying subjective, behavioral and psychophysiological correlates of subliminal phobic stimuli, and the consequent theoretical and therapeutic implications. Overall, the papers considered in this review—after being systematized and analyzed—provide new elements to develop a better theory of what specific phobias are, why they arise, how they maintain, and which are the best therapies to treat them. Subliminal exposure seems to represent a promising tool for the treatment of specific phobias, even if it shows some limits. From a methodological point of view, analysis of experimental procedures allowed us to find some relevant methodological parameters contributing to explain the contrasting results reported in the literature concerning skin conductance responses as reliable reactions elicited by subliminal phobic stimuli: only studies using intertrial interval (ITI) greater than to 20 s and stimulus duration > 20 ms successfully yielded skin conductance reactions to subliminal phobic stimuli (Figure 2).

Integrating the main evidence coming from the studies included in this review, we can draw a partially unexpected pattern of reactions induced by subliminal phobic stimuli. A phobic stimulus administered in subliminal paradigms is processed way more deeply than expected. In <33 ms (or, anyway, outside of awareness), a phobic subject distinguishes a phobic stimulus from a stimulus sharing similar perceptual features (e.g., a spider from a crab, in Sebastiani et al., 2011), and shows correlates of specific activations of both peripheral (e.g., skin conductance) and central (e.g., significant activation of amygdala) nervous system (see Table 3). Furthermore, brain activations in response to subliminal phobic stimuli are enhanced in cortical areas that previously were thought not to be so significantly involved, even as compared to supraliminal stimuli (Siegel et al., 2017).

Psychophysiological correlates and behavioral measures can be modulated by the subliminal exposure, but this is not reflected in a reduction of subjective distress when consciously facing the phobic stimulus (see Table 3). This dissociation implies that successful subliminal desensitization cannot be judged based on extinction eventually observed in psychophysiological and/or behavioral measures.

Improvements in facing a consciously perceived phobic stimulus—when assessed—suggest a more complex truth, as we show in the integrated defensive-cognitive therapeutic model proposed in section Toward an Integrated Defensive-Cognitive Therapeutic Model: subliminal and supraliminal exposure to phobic stimuli seems to affect two distinct systems (Figure 3), both of which should be considered to reach the best efficacy and acceptability of treatment. To our knowledge, this model represents the first proposal of a therapeutic approach that integrates classic treatments with evidence coming from the growing body of literature concerning exposure to subliminal phobic stimuli. Future researches should combine subliminal and supraliminal desensitization techniques to check if they complete each other.

## Data Availability Statement

The original contributions presented in the study are included in the article/Supplementary Material, further inquiries can be directed to the corresponding author/s.

## Author Contributions

SF and DM participated in all phases of the work. PH contributed in writing and reviewing the work. AZ contributed in reviewing the work. AG contributed in conceiving and reviewing the work. All authors contributed to the article and approved the submitted version.

## Conflict of Interest

The authors declare that the research was conducted in the absence of any commercial or financial relationships that could be construed as a potential conflict of interest.
