# Supplementary material for: Systematic Review of Studies on Subliminal Exposure to Phobic Stimuli: Integrating Therapeutic Models for Specific Phobias
Source: Front Neurosci. 2021 Jun 2;15:654170. doi: 10.3389/fnins.2021.654170 (PMC8206785; doi:10.3389/fnins.2021.654170)
Supplement: Supplementary file 1 [file Data_Sheet_1.PDF]

Supplementary Material

Systematic review of studies on subliminal exposure to phobic stimuli: integrating therapeutic models for specific phobias

Appendix 1: Keywords

- **Covert paradigms:**

  backward masking

  masking

  attentional blink

  subliminal

  under threshold

  under-threshold

  subthreshold

  sub-threshold

  covert

  covert stimuli

  covert stimulus

  ERP

  fear conditioning

  perceptual masking

  preattentive

  pre-attentive

  unconscious

  not conscious

  priming

  implicit

  continuous flash suppression

  flash suppression

- **Emotional/phobic correlates:**

  emotion

  emotions

  fear

  sadness

  disgust

  anger

  surprise

  trust

  distrust

  anticipation

  phobia

  threat

  startle

Supplementary Table 1: PRISMA Check-list

Section/topic	#	Checklist item	Reported on page #
TITLE			
Title	1	Identify the report as a systematic review, meta-analysis, or both.	1

ABSTRACT			
Structured summary	2	Provide a structured summary including, as applicable: background; objectives; data sources; study eligibility criteria, participants, and interventions; study appraisal and synthesis methods; results; limitations; conclusions and implications of key findings; systematic review registration number.	1
INTRODUCTION			
Rationale	3	Describe the rationale for the review in the context of what is already known.	1-2
Objectives	4	Provide an explicit statement of questions being addressed with reference to participants, interventions, comparisons, outcomes, and study design (PICOS).	2-3, Table 1
METHODS			
Protocol and registration	5	Indicate if a review protocol exists, if and where it can be accessed (e.g., Web address), and, if available, provide registration information including registration number.	3-4
Eligibility criteria	6	Specify study characteristics (e.g., PICOS, length of follow-up) and report characteristics (e.g., years considered, language, publication status) used as criteria for eligibility, giving rationale.	3, Table 1
Information sources	7	Describe all information sources (e.g., databases with dates of coverage, contact with study authors to identify additional studies) in the search and date last searched.	3-5

Search	8	Present full electronic search strategy for at least one database, including any limits used, such that it could be repeated.	Table 2, Appendix 1
Study selection	9	State the process for selecting studies (i.e., screening, eligibility, included in systematic review, and, if applicable, included in the meta-analysis).	5, Fig.1
Data collection process	10	Describe method of data extraction from reports (e.g., piloted forms, independently, in duplicate) and any processes for obtaining and confirming data from investigators.	3-5
Data items	11	List and define all variables for which data were sought (e.g., PICOS, funding sources) and any assumptions and simplifications made.	Table 1, Table 3
Risk of bias in individual studies	12	Describe methods used for assessing risk of bias of individual studies (including specification of whether this was done at the study or outcome level), and how this information is to be used in any data synthesis.	13, Supplementary Table 2, Supplementary Table 3
Summary measures	13	State the principal summary measures (e.g., risk ratio, difference in means).	Table 3
Synthesis of results	14	Describe the methods of handling data and combining results of studies, if done, including measures of consistency (e.g., I^2) for each meta-analysis.	15-17, Figure 3
Risk of bias	1	Specify any assessment of risk of bias that may affect the	13,

across studies	5	cumulative evidence (e.g., publication bias, selective reporting within studies).	Supplementary Table 2, Supplementary Table 3
Additional analyses	16	Describe methods of additional analyses (e.g., sensitivity or subgroup analyses, meta-regression), if done, indicating which were pre-specified.	N/A
RESULTS			
Study selection	17	Give numbers of studies screened, assessed for eligibility, and included in the review, with reasons for exclusions at each stage, ideally with a flow diagram.	Figure 1
Study characteristics	18	For each study, present characteristics for which data were extracted (e.g., study size, PICOS, follow-up period) and provide the citations.	Table 1, Table 3
Risk of bias within studies	19	Present data on risk of bias of each study and, if available, any outcome level assessment (see item 12).	Supplementary Table 2, Supplementary Table 3
Results of individual studies	20	For all outcomes considered (benefits or harms), present, for each study: (a) simple summary data for each intervention group (b) effect estimates and confidence intervals, ideally with a forest plot.	Table 3
Synthesis of results	21	Present results of each meta-analysis done, including confidence intervals and measures of consistency.	N/A

Risk of bias across studies	22	Present results of any assessment of risk of bias across studies (see Item 15).	13, Supplementary Table 2, Supplementary Table 3
Additional analysis	23	Give results of additional analyses, if done (e.g., sensitivity or subgroup analyses, meta-regression [see Item 16]).	N/A
DISCUSSION			
Summary of evidence	24	Summarize the main findings including the strength of evidence for each main outcome; consider their relevance to key groups (e.g., healthcare providers, users, and policy makers).	5-13, Table 3
Limitations	25	Discuss limitations at study and outcome level (e.g., risk of bias), and at review-level (e.g., incomplete retrieval of identified research, reporting bias).	13-21
Conclusions	26	Provide a general interpretation of the results in the context of other evidence, and implications for future research.	22-28
FUNDING			
Funding	27	Describe sources of funding for the systematic review and other support (e.g., supply of data); role of funders for the systematic review.	29

Risk of bias

Risk of bias and methodological validity of the studies included in the present systematic review were assessed independently by the first two authors (SF and DM) using two different tools: for within-subject studies, the Single-Case Reporting Guideline In Behavioral Interventions (SCRIBE) Statement (Tate et al., 2016a, b); for studies including a pre-post measure, a Quality Assessment Tool adapted from previously published systematic reviews (see Cummings et al., 2008). Disagreements between the reviewers were resolved by discussion with a third reviewer (AG). Both assessment tools stated that the quality of the considered studies ranged from medium to good (see Supplementary Table 3 and Supplementary Table 4), thus allowing their inclusion.

Supplementary Table 2. Risk of bias in within subject studies (SCRIBE check-list)

Supplementary Table 3. Risk of bias in pre-post studies
