# Supplementary material for: Systematic Review of Studies on Subliminal Exposure to Phobic Stimuli: Integrating Therapeutic Models for Specific Phobias
Source: Front Neurosci. 2021 Jun 2;15:654170. doi: 10.3389/fnins.2021.654170 (PMC8206785; doi:10.3389/fnins.2021.654170)
Supplement: Supplementary file 3 [file Data_Sheet_3.PDF]

| Study | 1 | 2 | 3 | 4 | 5 | 6 | 7 | 8 | 9 | 10 | 11 | 12 | 13 | 14 | 15 | 16 | 17 | 18 | 19 | 20 | 21 | 22 | 23 | 24 | 25 | 26 |
|---|---|---|---|---|---|---|---|---|---|---|---|---|---|---|---|---|---|---|---|---|---|---|---|---|---|---|
| Öhman & Soares, 1994 | no | yes | yes | yes | yes | no | no | yes | yes | yes | yes | yes | no | yes | yes | yes | no | yes | no | no | yes | yes | no | no | no | no |
| Merckelbach et al., 1995 | no | yes | yes | yes | yes | no | yes | yes | yes | yes | yes | yes | no | yes | yes | yes | no | yes | no | no | no | yes | yes | no | no | no |
| van den Hout et al., 1997 | no | yes | yes | yes | yes | no | no | yes | yes | yes | yes | yes | no | yes | yes | yes | no | yes | no | no | yes | yes | no | no | no | no |
| Thorpe & Salkovskis, 1997 | no | yes | yes | yes | yes | no | no | yes | yes | yes | yes | yes | no | yes | yes | yes | no | yes | no | no | no | yes | yes | no | no | yes |
| Mayer et al., 1999a | no | yes | yes | yes | yes | no | no | yes | yes | yes | yes | yes | no | yes | yes | yes | no | yes | no | no | no | yes | yes | no | no | no |
| van den Hout et al., 2000 | no | yes | yes | yes | yes | no | no | yes | yes | yes | yes | yes | no | yes | yes | no | no | yes | no | no | yes | yes | yes | no | no | no |
| Wikström et al., 2004 | no | yes | yes | yes | yes | no | no | yes | yes | yes | yes | yes | no | yes | yes | yes | no | yes | no | no | no | yes | yes | no | no | no |
| Carlsson et al., 2004 | no | yes | yes | yes | yes | no | no | yes | yes | yes | yes | yes | yes | yes | yes | yes | no | yes | no | no | no | yes | yes | no | no | no |
| Carretié et al., 2005 | no | yes | yes | yes | yes | no | no | yes | yes | yes | yes | yes | no | yes | yes | yes | no | yes | no | no | no | yes | yes | no | no | yes |
| Ruiz-Padial et al., 2005 | no | yes | yes | yes | yes | no | no | yes | yes | yes | yes | yes | no | yes | yes | yes | no | yes | no | no | yes | yes | yes | no | no | yes |
| Granado et al., 2007 | no | yes | yes | yes | yes | no | no | yes | yes | yes | yes | yes | yes | yes | yes | yes | no | yes | no | no | yes | yes | yes | yes | no | yes |
| Siegel & Weinberger, 2009 | no | yes | yes | yes | yes | yes | no | yes | yes | yes | yes | yes | yes | yes | yes | yes | no | yes | no | no | no | yes | yes | yes | no | no |
| Weinberger et al., 2011 | no | yes | yes | yes | yes | yes | no | yes | yes | yes | yes | yes | yes | yes | yes | yes | no | yes | no | no | no | yes | yes | yes | no | no |
| Siegel & Weinberger, 2012 | no | yes | yes | yes | yes | yes | yes | yes | yes | yes | yes | yes | yes | yes | yes | yes | no | yes | no | no | no | yes | yes | yes | no | no |
| Sebastiani et al., 2011 | no | yes | yes | yes | yes | no | no | yes | yes | yes | yes | yes | yes | yes | yes | yes | no | yes | no | no | no | yes | yes | yes | no | yes |
| Siegel et al., 2011 | no | yes | yes | yes | yes | yes | yes | yes | yes | yes | yes | yes | yes | yes | yes | yes | no | yes | no | no | no | yes | no | yes | no | no |
| Lipka et al., 2011 | no | yes | yes | yes | yes | no | no | yes | yes | yes | yes | yes | yes | yes | yes | yes | no | yes | no | no | no | yes | yes | yes | no | yes |
| Peira et al., 2012 | no | yes | yes | yes | yes | no | no | yes | yes | yes | yes | yes | yes | yes | yes | yes | no | yes | no | no | no | yes | yes | yes | no | no |
| Gutner et al., 2012 | no | yes | yes | yes | yes | no | no | yes | yes | yes | yes | yes | yes | yes | yes | yes | no | yes | no | no | yes | yes | yes | yes | no | no |
| Siegel & Warren, 2013 | no | yes | yes | yes | yes | no | no | yes | yes | yes | yes | yes | yes | yes | yes | yes | no | yes | no | no | no | yes | yes | yes | no | no |
| Lipka et al., 2014 | no | yes | yes | yes | yes | no | no | yes | yes | yes | yes | yes | yes | yes | yes | yes | no | yes | no | no | no | yes | yes | yes | no | yes |
| Siegel & Gallagher, 2015 | no | yes | yes | yes | yes | no | no | yes | yes | yes | yes | yes | no | yes | yes | yes | no | yes | no | no | yes | yes | yes | yes | no | no |
| Schmack et al., 2016 | no | yes | yes | yes | yes | no | no | yes | yes | yes | yes | yes | yes | yes | yes | yes | no | yes | no | no | yes | yes | yes | yes | no | yes |
| Siegel et al., 2017 | no | yes | yes | yes | yes | no | no | yes | yes | yes | yes | yes | yes | yes | yes | yes | no | yes | no | no | yes | yes | yes | yes | no | yes |
| Siegel et al., 2018 | no | yes | yes | yes | yes | no | yes | yes | yes | yes | yes | yes | no | yes | yes | yes | no | yes | no | no | no | yes | yes | yes | no | no |
| Taschereau-Dumouchel et al., 2018 | no | yes | yes | yes | yes | yes | no | yes | yes | yes | yes | yes | yes | yes | yes | yes | no | yes | no | no | yes | yes | yes | yes | no | yes |
| **Study** | **1** | **2** | **3** | **4** | **5** | **6** | **7** | **8** | **9** | **10** | **11** | **12** | **13** | **14** | **15** | **16** | **17** | **18** | **19** | **20** | **21** | **22** | **23** | **24** | **25** | **26** |

1. *Title* identify the research as a single-case experimental design in the title
2. *Abstract* summarize the research question, population, design, methods, including intervention/s (independent variable/s) and targe behavior/s and any other outcome/s (dependent variable/s), results, and conclusions
3. *Scientific background* describe the scientific background to identify issue/s under analysis, current scientific knowledge, and gaps in that knowledge base
4. *Aims* state the purpose/aims of the study, research question/s, and, if applicable, hypotheses
5. *Design* identify the design (e.g., withdrawal/reversal, multiple-baseline, alternating-treatments, changing-criterion, some combination thereof, or adaptive design) and describe the phases and phase sequence (whether determined a priori or data-driven) and, if applicable, criteria for phase change
6. *Procedural changes* describe any procedural changes that occurred during the course of the investigation after the start of the study
7. *Replication* describe any planned replication
8. *Randomization* state whether randomization was used, and if so, describe the randomization method and the elements of the study that were randomized
9. *Blinding* state whether blinding/masking was used, and if so, describe who was blinded/masked
10. *Selection criteria* state the inclusion and exclusion criteria, if applicable, and the method of recruitment
11. *Participant characteristics* for each participant, describe the demographic characteristics and clinical (or other) features relevant to the research question, such that anonymity is ensured
12. *Setting* describe characteristics of the setting and location where the study was conducted
13. *Ethics* state whether ethics approval was obtained and indicate if and how informed consent and/or assent were obtained
14. *Measures* operationally define all target behaviors and outcome measures, describe reliability and validity, state how they were selected, and how and when they were measured
15. *Equipment* clearly describe any equipment and/or materials (e.g., technological aids, biofeedback, computer programs, intervention manuals or other material resources) used to measure target behavior/s and other outcome/s or deliver the interventions
16. *Intervention* describe the intervention and control condition in each phase, including how and when they were actually administered, with as much detail as possible to facilitate attempts at replication
17. *Procedural fidelity* describe how procedural fidelity was evaluated in each phase
18. *Analyses* describe and justify all methods used to analyze data
19. *Sequence completed* for each participant, report the sequence actually completed, including the number of trials for each session for each case. For participant/s who did not complete, state when they stopped and the reasons
20. *Outcomes and estimation* for each participant, report results, including raw data, for each target behavior and other outcome/s
21. *Adverse events* state whether or not any adverse events occurred for any participant and the phase in which they occurred
22. *Interpretation* summarize findings and interpret the results in the context of current evidence
23. *Limitations* discuss limitations, addressing sources of potential bias and imprecision
24. *Applicability* discuss applicability and implications of the study findings
25. *Protocol* if available, state where a study protocol can be accessed
26. *Funding* identify source/s of funding and other support; describe the role of funders
